# Statistical modeling of numbers of human deaths per road traffic accident in the Oromia region, Ethiopia

**DOI:** 10.1371/journal.pone.0251492

**Published:** 2021-05-19

**Authors:** Merga Abdissa Aga, Berhanu Teshome Woldeamanuel, Mekonnen Tadesse

**Affiliations:** 1 Department of Statistics, Salale University, Oromia, Ethiopia; 2 Department of Statistics, Addis Ababa University, Addis Ababa, Ethiopia; Tsinghua University, CHINA

## Abstract

**Background:**

Globally, road traffic accidents are the leading causes of death among young people in general, and the main cause of death among young people aged 15–29 years. Recently, in Ethiopia, the number of road traffic accidents has been increasing. The study aimed to identify the potential factors associated with the number of human deaths by road traffic accidents in the Oromia Regional State, Ethiopia.

**Methods:**

We used data obtained from the Oromia region traffic police office recorded on daily basis road traffic accidents from July 2016 up to July 2017. Count regression models were was used to analyses the factors associated with the number of human deaths from road traffic accidents.

**Results:**

Age of the driver’s 31–50 years (AOR = 0.289, 95%CI: 0.175, 0.479) and higher than 50 years old (AOR = 0.311, 95%CI: 0.129, 0.751), driver’s years of experience 5–10 years (AOR = 0.014, 95%CI: 0.007, 0.027), and more than 10 years (AOR = 0.101, 95%CI: 0.057, 0.176), automobile vehicle type (AOR = 8.642, 95%CI: 2.7644, 27.023), vehicle years of service 5–10 years (AOR = 2.484, 95%CI: 1.194, 5.169), and more than 10 years (AOR = 2.639, 95%CI: 1.268, 5.497), vehicle upside down accidents (AOR = 5.560, 95%CI: 2.506, 12.336), turning illegal position (AOR = 0.454, 95%CI: 0.226, 0.913), residential areas (AOR = 108.506, 95%CI: 13.725, 857.798), and working areas (AOR = 129.606, 95%CI: 16.448, 1021.263) were significant associated number of human deaths per road traffic accident factors in the study area.

**Conclusion:**

Human deaths per road traffic accidents occurred due to the younger age of the driver, driver’s lack of sufficient experience, vehicle serviced for long years, driving on a wet road, driving in the afternoon, driving near/around residential places and vehicle to driver’s relation. Thus, the regional traffic police should give special attention to younger drivers, less experienced drivers, old vehicles, driving near residential areas, driving automobiles, and driving in the afternoon to control traffic system to reduce the number of human deaths pear road traffic accident.

## Background

Road traffic accidents (RTA) constitute a major public health and development crisis globally. Over 1.2 million people die each year worldwide due to road traffic accidents [1], with millions suffering serious injuries and living with long-term adverse health consequences. Road traffic accidents are a leading cause of death, particularly among young people, aged 15–29 years [2]. Even though small numbers of vehicles the vast majority of road traffic deaths happened in low-income countries. More than 90% of road traffic deaths occur in low and middle-income countries, yet these countries have just 54% of the world’s vehicles [3].

Road traffic accidents place a heavy burden on national economies as well as on households. In low and middle-income regions they predominantly affect the economically energetic age group, or those set to subsidize the household and the labor force in general. Numerous families are driven further into neediness by the passing of a provider, or by the expenses of extended clinical consideration, or the additional weight of thinking about a relative who is crippled due to street traffic injury. The monetary expenses also strike hard from one side of the country to the other, forcing a critical weight on wellbeing, protection, and lawful frameworks. This is transcendently verifiable in countries battling with other improvement needs, where interest in street security isn’t proportionate with the size of the issue. Data suggest that road traffic deaths and injuries cause economic losses up to 3% of gross domestic product (GDP) globally, whereas in low and middle-income countries they are estimated at 5% of GDP [3].

The African countries remain to have the highest road traffic death rates. The situation of road traffic accidents is most severe in sub-Saharan Africa, where the lives of millions are lost and a significant amount of property is damaged. In Ethiopia, the condition has been crumbled as the number of vehicles has risen and accordingly because of expanded traffic stream and experiences among vehicles and people on foot. Despite government efforts in road development, road accidents remain to be one of the critical problems of the road transport sector in Ethiopia [4]. Every year, many lives are lost and much property is destroyed due to road traffic accidents in the country. The country has experienced average annual road accidents of 8115 during 2000–2010 [5].

Among many low-income nations in Africa; Ethiopia has a high rate of road traffic injury and passing [6]. Road accident in Ethiopia is one of the most exceedingly terrible accident records in the world, as communicated per 10,000 vehicles. Moreover, road accidents are concentrated in Addis Ababa, which is the capital city of Ethiopia and Oromia regional State, representing 58% of all deaths and two-third of all injuries [7]. During 2006–2015, the average number of road traffic accidents expanded in Ethiopia, where Oromia regional, state represents most of the complete fatalities happened and Addis Ababa city administration represents the major genuine and slight injuries just as property harms [8].

According to [8], Road traffic accident in Ethiopia is showing a rising trend over the last decades. Moreover, higher road accidents are concentrated in Oromia regional State and Addis Ababa. Not only the traffic accidents in Ethiopia are concentrated in the Oromia region and Addis Ababa city, but also the volume of motorized traffic is very high as compared to the other regions of the country.

Previous studies conducted on road traffic accident focused on Addis Ababa city survey of road traffic accidents. Further, these investigations utilized descriptive analysis, logistic regression, and Poisson regression models for data analysis [9–13]. In the investigation of the number of human fatalities, count regression models are more fitting than other methods. Many prior researchers categorized the count variable as binary, but this may underestimate the total number of deaths due to road traffic accidents since multiple human deaths are collapsed into a single unit.

Ethiopia is one of the developing countries with a low level of income coupled with a high rate of population growth, where Oromia is the largest region in the country with a total population estimated more than 37 Million [14]. To extend our comprehension of the most well-known and steady factors on the risk of human death due to road traffic accidents, this study has identified the human, environmental, road, and vehicle characteristics associated with human death by road traffic accidents in Oromia regional state of Ethiopia. Moreover, to the best of our knowledge, none of these researchers use zero-inflated models and other higher count regression models. Therefore, this study aims to identify the major factors determining the number of human deaths per road traffic accident in the Oromia Regional State, Ethiopia via the appropriate count regression models.

## Methods

### Study setting and design

This study was based on a retrospective study of all registered road traffic accidents in the Oromia region from July 2016 up to July 2017. Secondary data from the Oromia regional state traffic police office registers were taken. 3900recorded RTA was included in the study. The Oromia regional state was one of the nine regions in Ethiopia, which is the largest region of the country. The major towns of the region include Adama, Ambo, Asella, Bale Robe, Bishoftu, Fiche, Goba, Jimma, Metu, Nekemte, Sebeta, Sululta, Shashemene, and Wolliso among many others [15]. The Oromia region has the highest traffic movement in the country, next to Addis Ababa. The major road from Addis Ababa to Djibouti passes through this region. All major roads between Addis Ababa city and other regions’ capital cities will pass this region.

### Data collection

The secondary data were obtained from Oromia region traffic police control and investigation offices records using a checklist that was prepared based on the road traffic control and registry format. The collected data were tested for completeness. Then, cleaning, editing, and eliminating missing values were done using STATA version 13 software.

### Variables of the study

The response variable of this study was the number of human deaths per road traffic accident in the Oromia region measured as 0, 1, 2, 3…A human death includes pedestrians, road users, passengers, or other people’s death due to road traffic accident. The potential predictors related to the number of human deaths per road traffic accident adopted from various literature [16–22] were demographic characteristics (sex and age of driver), environmental characteristics (time of the accident, road condition, the environment of the accident, weather condition, location of the accident, day of the accident), vehicle-related characteristics (vehicle type, vehicle service years), road pavement, driving experience, education level of the driver, driver-vehicle relationship, accident cause, and accident type (collision between vehicles, vehicle, and pedestrian, vehicle and animal, or vehicle upside down).

#### Statistical data analysis

We used STATA version 13 [23] to analyze data. We illustrated the distribution of data using tables, percentages, mean, variance, skewness, and kurtosis. Count regression models were used as a method to model the discrete nature of the response variable (number of human deaths per road traffic accidents) measured as 0, 1, 2, 3… [24,25].

Count regression models, unlike linear regression, have counted response variable that can take only non-negative integer values, 0, 1, 2, 3… measured in natural units on a fixed scale representing the number of times an event (number of human deaths per road traffic accident) occurs in a fixed domain. For count data, the standard framework for explaining the relationship between the outcome variable and a set of predictors includes the standard count regression models (Poisson Regression and Negative Binomial (NB) Regression), Zero-inflated models (Zero-inflated Poisson (ZIP) and Zero-inflated Negative Binomial (ZINB)) and Hurdle models (Hurdle Poisson (HP) and Hurdle Negative binomial (HNB)) were used.

The conventional Poisson regression model for count data is often of limited use because the empirical count data set typically exhibit over-dispersion and/or have an excess number of zeros. This problem can be addressed by extending the ordinary Poisson regression model by including the dispersion term in the model. The family of generalized linear models (GLMs) negative binomial regression model was the solution [26–28] However, even though these models normally can catch over-dispersion rather well, they are in many applications not adequate for modeling overabundance zeros. Zero-augmented models were addressing this issue by capturing zero counts [29,30]. Conversely, Zero-inflated models [29], are mixture models that combine a count component and a point mass at zero. A comprehensive and up-to-date account of count models and methods as well as the interpretations of fitted count models are provided by [31–35]. Hurdle models [30,36–38] combine a left-truncated count component with a right-censored hurdle component.

Let y_i_ represent counts of events (number of deaths) occurring of the random variable Y in a given time or exposure periods with rate μ and the vector x′ = [*x*_*1*_, *x*_*2*_,*…*, *x*_*p*_] a group of *p* predictors variables. Then the probability mass function for a Poison random variable Y is given by;

$$p(Y=y_{i}|\mu )=\frac{e^{-\mu }\mu^{y_{i}}}{y_{i}!},\;\mu \geq 0,\;y_{i}=0,1,2,\ldots$$ (1)

where *y*_i_ = 0, 1, 2, 3… are discrete counts (the number of human ddeathsper accident) and μ is the rate parameter [29].

Then the relationship between the predictors the non-negative mean parameter μ_i_ is the exponential specification given by;

$$E\left(y_{i}\right)=\mu_{i}=\exp\;(x_{i}\prime \beta )$$

where *x*_i_′ = (1, *x*_*i*1_, …, *x*_*ip*_), is a vector of explanatory variables and *β* = (*β*_0_, *β*_1_, *β*_2_, …, *β*_*p*_)′ is the corresponding (*p* + 1) dimensional column vector of unknown parameters to be estimated.

The unknown parameters of the model were estimated using the maximum likelihood estimation of the log-likelihood function.

Given that over-dispersion is the norm, the negative binomial model has more generality than the Poisson model. Over-dispersion is most often caused by the highly skewed response/dependent variables or often due to variables with high numbers of zeros [25,30,31]. The probability mass function of a negative binomial distribution random variable Y is given by;

$$p(y_{i}|\mu ,\delta )=\frac{\Gamma \left(y_{i}+\frac{1}{\delta }\right)}{y_{i}!\Gamma \left(\frac{1}{\delta }\right)}{\left(1+\delta \mu \right)}^{\frac{1}{\delta }}{\left(1+\frac{1}{\delta \mu }\right)}^{-y_{i}},\;y_{i}\geq 0,\;\delta >0$$ (2)

The mean and variance of NB distribution are E(*y*|μ,δ) = μ, and *va*r(*y*|μ,δ) = μ(1 + δμ). Where δ is the dispersion parameter [29]. The predictor variables related to the parameter μ through the log-link function defined as logμ = *x*_i_′β.

In the real-life data, the major source of over-dispersion is a relatively large number of zero counts and the resulting over-dispersion cannot be modeled accurately with the negative binomial model [34,35]. In such cases, one may use zero-inflated models [27] that are a mixture of two separate data generation processes: one generates only zeros, and the other is either a Poisson or negative binomial data-generating process are immediate solution.

In ZIP models, the underlying Poisson distribution for the first subpopulation is assumed to have a variance that is equal to the distribution’s mean. If this is an invalid assumption, the data exhibit overdispersion (or under dispersion). The probability distribution of a zero-inflated Poisson random variable is given by:

$$p(y_{i}|\mu_{i})=\{\begin{array}{l} \omega_{i}+(1-\omega_{i})e^{-\mu_{i}},\;y_{i}=0\\ (1-\omega_{i})\frac{e^{-\mu_{i}}\mu_{i}{}^{y_{i}}}{y_{i}!},\;y_{i}=1,2,3,\ldots ,\;0\leq \omega_{i}\leq 1 \end{array}$$ (3)

The response variable y_i_ is a non-negative integer, μ_i_ is the expected Poisson count for the i^th^ individual; ω_i_ is the probability of extra zeros. The mean and variance of ZIP distribution are E(Y_i_) = (1 − ω_i_) μ_i_ and υar(Y_i_) = *E*(Y_i_)(1 + ω_i_μ_i_). The parameters μ_i_ and ω_i_ depend on covariates xi and z_i_ respectively, where log(μ_i_) = x_i_^’^β and log ($\frac{\omega i}{1-\omega i}$) = z_i_^’^γ

Furthermore, the theory suggests that the excess zeros are generated by a separate process from the count values and that the excess zeros can be modeled independently. The probability distribution of a zero-inflated negative binomial response variable is given by:

$$p(y_{i}|\mu_{i},\omega_{i})=\{\begin{array}{l} \omega_{i}+(1-\omega_{i}){\left(1+\delta \mu_{i}\right)}^{-\frac{1}{\delta }},\;y_{i}=0\\ (1-\omega_{i})\frac{\Gamma \left(y_{i}+\frac{1}{\delta }\right)}{y_{i}!\Gamma \left(\frac{1}{\delta }\right)}{\left(1+\delta \mu_{i}\right)}^{-\frac{1}{\delta }}{\left(1+\frac{1}{\delta \mu_{i}}\right)}^{-y_{i}},\;y_{i}>0 \end{array}$$ (4)

where δ > 0 is an over-dispersion parameter. The mean and variance of the ZINB model are E(*Y*_i_) = (1 − ω_i_) μ_i_ and *V*ar(*Y*_i_) = (1 − ω_i_) (1 + ω_i_μ_i_ + δμ_i_) μ_i_. The parameters μ_i_ and ω_i_ depend on vectors of covariates x′ = [x_1_, x_2_, …, x_p_] and *z*_i_, respectively. The method of Fisher scoring is more appropriate to obtain the parameter estimates of ZINB regression models.

Hurdle count models are two-component models with a truncated count component for positive counts and a hurdle component that models the zero counts. The count model is typically a truncated Poisson or negative binomial regression (with log link) [24,25,37,38]. The probability mass function of the response variable y in the hurdle model is given by:

$$p(y=y_{i}/x_{i},\;z_{i},\;\beta ,\gamma )=\left\{ \begin{array}{c} f_{zero}(0;\;z_{i};\;\gamma )\;if\;y_{i}=0\\ (1-f_{zero}(0;\;z_{i};\;\gamma ))(\frac{f_{count}}{\left(1-f_{count}(0;x_{i};Ɓ\right)} \end{array}\right.(y_{i};\;x_{i};\;\beta ))\;if\;y_{i}>0$$ (5)

where *y*_*i*_ is the value of the dependent variable for the *i*^*th*^ person *i =* 1, …, *n*), *z*_*i*_ is a vector denoting the number of predictor variables in the zero counts, *x*_i_ represents a vector denoting the number of predictor variables in the hurdle part, *γ* is a vector of coefficients belonging to *z*, and *β* denotes a vector of coefficients related to *x*, *f zero* is a probability density function at least binary outcome (0, 1) or counts (0, 1, 2, 3…), and *f count* is a probability density function of counts (0, 1, 2, 3 …).

The *f*_*zero*_ part, where *y*_*i*_ *=* 0 is typically modeled with a binary logit (logistic regression) model, where all counts greater than 0 are given a value of one. Using a binary logistic regression model for this part, the probability of *y*_*i*_ = 0 is denoted as;

$$f_{zero}(0;\;z_{i};\;\gamma )=\psi_{i}=\frac{1}{1+e^{z_{i}\gamma }}$$

where *z*_*i*_ represents the observed data and *γ* the vector of coefficients belonging to *z*_*i*_. The probability of a nonzero count is given by 1 –*ψ*_*i*_. The non-zero count part *(fcount)* is modeled with a truncated (y_i_ > 0) count model [37–39].

The model selection criterion was based on the Log pseudo likelihood, Akaike information criterion (AIC) and Bayesian information criterion (BIC). A smaller Log pseudo likelihood, AIC, and BIC value suggest that the model is of better fit [40]. Furthermore, the Vuong statistic was used to compare the ZIP versus the Poisson model and ZINB versus the NB model for predicting human death per accident [40,41]. The Deviance and Pearson Chi-square statistics were used for testing overall model goodness of fit [42]

## Results

### Descriptive statistics

**Fig 1:**
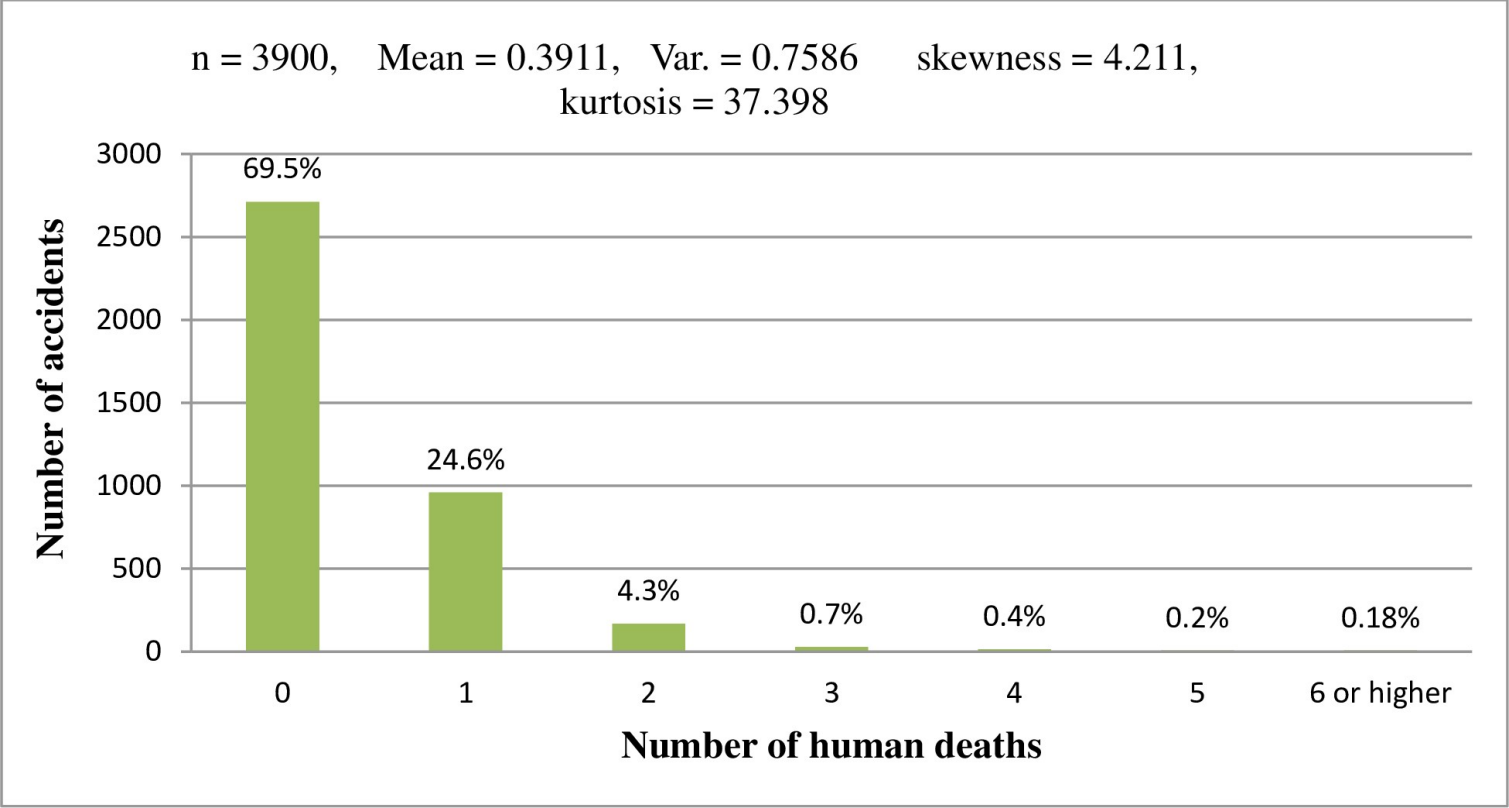
Bar chart of distribution of response variable, count of human death per road traffic accidents.

A total of 3, 900 road traffic accidents happened in the study period, of which 1,188 accidents involved the death of 1,541 people. As shown in Fig 1, the variance of the number of human deaths per accident is greater than its mean, suggesting a possibility of over-dispersion. About 69% of the accidents were nonfatal; suggesting excess zeros in the dataset. Further, the bar chart in Fig 1 is highly skewed to the right showing massive counts of zero outcomes. However, a large number of human deaths per accident were less frequently observed, implying that dual regime event count models such as zero-inflated models and hurdle models will often tend to indicate over-dispersion in the data as a result of a large number of zero counts (Fig 1).

The vast majority (92.4%) of study participants were male drivers and about half (50.9%) were in the age range between 18 and 30 years old. Nearly three-fourths (71.2%) of the drivers had primary school or lower and only one-tenth (11%) were attained higher education. Regarding driving experience, more than two-thirds (69.2%) had only five or lower years of driving experience, while only 12.9% had at least 10 years of driving experience. Similarly, as a driver to vehicle relationship was concerned 86% were employed drivers. Most of the accidents were due to not keeping distance between while driving and not given priority to pedestrians. On the other hand, when the type of accident was concerned, the majority (51.4%) were reported a collision between vehicles. Table 1 reveals that about 60% of the accidents happened in the central zone of the region. It also indicates that 58.9% of vehicles have five or lower years of services.

**Table 1:** Descriptive statistics RTA by predictor variables.

Predictor variables	Categories	n	percent	Mean	Variance
Gender of the Driver	Male	3605	92.44	0.3953	0.5066
	Female	295	7.56	0.3932	1.4231
Age of Driver	18–30	1986	50.92	0.4874	0.9109
	31–50	1121	28.74	0.3568	0.2297
	51 and above	793	20.33	0.2181	0.1707
Driving Experience	Less than 5 years	2696	69.18	0.4362	0.7325
	5–10 years	698	17.90	0.3510	0.2281
	Above 10 years	504	12.92	0.2361	0.1807
Vehicle driver relationship	Employee	3355	86.03	0.4399	0.6364
	Owner	379	9.72	0.1160	0.1028
	Others	166	4.26	0.3072	0.2141
Vehicle Type	Taxi-minibuses (up to 12 Seats)	1457	37.36	0.1983	0.1591
	Automobile	90	2.31	0.5555	0.2497
	Pick up (up to 10 quintals)	375	9.62	0.2960	0.2089
	Cargo (11–40 quintals)	416	10.67	0.2764	0.2005
	Cargo (41–100 quintals)	284	7.28	0.3838	0.2373
	Cargo with trailer (up to 400 quintals)	503	12.90	0.3837	0.2369
	Buses (13–45 seats)	290	7.44	1.2517	1.1094
	Buses (above 46 seats)	114	2.92	0.9824	3.9288
	Others	371	9.51	0.5364	1.4872
Vehicle service	Up to 5 years	2296	58.87	0.3649	0.2318
	6–10 years	927	23.77	0.3204	0.4058
	Above 10 years	677	17.36	0.5997	1.928
Road Condition	Wet	1817	46.59	0.4259	0.6863
	Dry	414	10.62	0.4082	0.2421
	Muddy	1669	42.79	0.3583	0.5358
Weather Condition	Not normal (cloudy, rainy, cold, hot)	3432	88.0	0.4146	0.6252
	Normal	468	12.0	0.2521	0.1889
Accident Time	Afternoon	1309	33.56	0.4553	0.9928
	Morning	705	18.08	0.3049	0.2122
	Evening	1303	33.41	0.4029	0.3728
	Night	583	14.95	0.3516	0.5136
Location of the Accident	Central zones	2371	60.79	0.4496	0.7960
	Western zones	407	10.44	0.2113	0.1670
	Southwest zones	311	7.97	0.3633	0.2320
	Easter Zones	359	9.21	0.4011	0.2409
	Southeast zones	452	11.59	0.2920	0.2338
Environment of the Accident	Factory place	303	7.77	0.1320	0.1149
	Residence place	447	11.46	0.8009	2.6037
	Commercial place	491	12.59	0.4562	0.2486
	Religious place	340	8.72	0.3471	0.2273
	Entertainment place	355	9.10	0.3662	0.2327
	School place	361	9.26	0.2770	0.2008
	Hospital place	325	8.33	0.3323	0.2225
	In village rural area	454	11.64	0.2378	0.1817
	Out of village in a rural area	442	11.33	0.2262	0.1754
	Government office place	382	9.79	0.6675	0.8052
Accident Type	Collision between Vehicles	2004	51.38	0.4361	0.9011
	Collision between vehicle and pedestrian	341	8.74	0.5366	0.2494
	Collision between vehicle and animal	201	5.15	0.4826	0.2509
	vehicle upside down	769	19.72	0.2145	0.1687
	Others	585	15.00	0.3795	0.2358
Educational level of the driver	Elementary school and below	2778	71.23	0.4449	0.6885
	High School	679	17.41	0.4123	0.3695
	Above high school	443	11.36	0.0564	0.0533
Day of the weeks	Monday	659	16.90	0.3050	0.2123
	Tuesday	557	14.28	0.3052	0.2124
	Wednesday	529	13.56	0.3043	0.2121
	Thursday	574	14.72	0.3048	0.2123
	Friday	495	12.69	0.3050	0.2124
	Saturday	640	16.41	0.6547	1.7788
	Sunday	446	11.44	0.5919	0.9612
Road pavement	Good Asphalt	1405	36.03	0.3388	0.2242
	Defected Asphalt	1220	31.28	0.3877	0.7691
	Gravel	1152	29.54	0.4392	0.7174
	Site clearing	123	3.15	0.6992	1.2120
Road inclination	Not straight(sloped, curved, scarp, uphill)	3416	87.59	0.4124	0.6265
	Straight	484	12.41	0.2727	0.1987
Accident cause	not given priority to pedestrian	525	13.46	0.7085	0.2068
	Overload	222	5.69	0.0990	0.0896
	Over speed	246	6.31	0.0203	0.0199
	Turning an illegal position	480	12.31	0.2229	0.1736
	Steering problem	580	14.87	0.8034	2.2272
	Brake problem	546	14.00	0.3095	0.2141
	Release of tyre	614	15.74	0.3306	0.4076
	following not keeping distance between	687	17.62	0.2867	0.2048

Table 1 shows that the mean number of human deaths per accident was almost similar between male and female drivers. The average number of human deaths per accident was inversely related to driver’s age, the highest average number of human deaths per accident (mean = 0.4874) was seen among the youngest age group drivers (18–30 years). Considering the experience of the driver, the highest mean number of human deaths occurred among drivers with less than 5 years of driving experience (mean = 0.4362), while the smallest (mean = 0.2361) was reported among drivers with more than 10 years of driving experience. Employed drivers had the highest mean number of human deaths (mean = 0.4399); while the smallest was among owner-drivers (mean = 0.1161). Considering the vehicle type, the highest mean number of human deaths (mean = 1.2517) was attributable to buses with 13–45 seats, whilst the smallest (mean = 0.1983) was due to minibusses and automobiles. Although it showed that the highest vehicle service years (10 years or above), was related to the highest mean number of human deaths per road traffic accidents (mean = 0.5997).

Concerning the accident time, the highest mean number of human deaths per road traffic accident occurs in the evening time. On the other hand, regarding the geographical distribution, the highest mean number of human deaths per accident occurred in central zones (special zones surrounding Finfinne and, Shoa zones mean = 0.4496), whereas the lowest was occurred in the western zones of the region (mean = 0.2113). Furthermore, the highest mean number of human death was happening near residential areas. As a type of accident was concerned, accident collision between vehicle and pedestrian had the highest mean number of human deaths (mean = 0.5366), followed by a collision between vehicles and animals (mean = 0.4826), and collision between vehicles (mean = 0.4361). Comparatively, accidents that turned vehicles upside down had the smallest mean number of human deaths per accident (mean = 0.2145).

Regarding the educational level of drivers, the highest mean number of human deaths (mean = 0.4449) was reported among drivers with primary education, while the smallest (mean = 0.0564) was seen among drivers having more than high school. Moreover, the highest mean number of human deaths per accident occurred during the weekend as compared to the other days. The highest mean number of human deaths per accident happened on-site clearing roads and gravel roads as compared to the other road pavements such as asphalt roads. Similarly, a higher mean number of human deaths happened on roads that were sloppy, curved, sharp, and uphill than straight roads. Concerning the causes of accidents, the highest mean number of human deaths occurred due to the steering problem (mean = 0.8034) followed by drivers denying priority to pedestrians (mean = 0.7085). On the contrary, over-speed and overload accounted for the lowest mean number of human deaths. Whereas turning to illegal direction, brake problem, the respire of the tire and not keeping the minimum required distance between had an intermediate mean human death per accident.

### Test of over-dispersion, goodness-of-fit test, and comparison

Initially, we fitted the Poison model. Then the fitted Poison model was tested for over-dispersion. It was found that the over-dispersion parameter is significant (deviance statistics = 4060.98, p-value = 0.035), (Pearson Chi-square = 5679.44, p-value < 0.0001). Further, the six different models namely; Poisson, NB, ZIP and ZINB, HP, and HNB were compared using Log pseudo-likelihood, AIC, and BIC to identify an appropriate model that fits the data well. Moreover, the Vuong statistic (Voung = 27.74, p-values < 0.0001) for comparing the ZIP versus Poisson model was significant, implying that the ZIP model is preferred to the Poisson model for predicting the human death per accident. Likewise, the calculated value of the Vuong test statistic for comparing ZINB versus NB models (Voung = 28.45, p-values < 0.0001) was significant, indicating that the ZINB model is preferred to the NB regression model. Finally, the Log pseudo-likelihood, AIC, and BIC found that the HP model was the most appropriate model to fit the number of human deaths per road traffic accident than other models since it has the smallest AIC, BIC, and maximum log pseudo-likelihood (Table 2).

**Table 2:** Model selection and fit statistics for model comparison.

Selection criteria	Models					
	Poisson	NB	ZIP	ZINB	HP	HNB
Logpseudo likelihood	-1616.283	-1571.679	-1307.637	-1395.554	**-914.64**	-1219.76
AIC	3400.567	3331.359	2759.279	2989.108	**2037.28**	2649.52
BIC	3927.14	3920.62	3210.623	3609.713	**2627.41**	3144.73
Vuong test (p-value)			27.74 (< 0.0001)	28.45(< 0.0001)		

### Factors associated with the number of human deaths per road traffic accidents

The estimated Hurdle Poison regression model was presented in Table 3. The model has two parts. The first part is from the equation predicting counts for numbers of human death per road traffic accidents (truncated Poisson with log link). The second part of the model predicts the zero hurdle model (binomial with logit link) zero deaths versus not zero deaths.

**Table 3:** Factors associated with the number of human deaths per road traffic accident, Hurdle Poison model.

Variables	Count Model coefficients (truncated Poisson with log link)			Zero hurdle model (binomial with logit link)		
	Odds ratio	95% CI for Odds ratio	p-values	Odds ratio	95% CI for Odds ratio	p-values
Gender of driver						
Male	1					
Female	1.113	0.969, 1.28	0.1290	0.132	0.0008, 20.918	0.4340
Age of driver						
18–30	1					
31–50	0.289**	0.175, 0.479	< 0.0001	11658.88	4.891E-07, 2.78E+14	0.4420
51 and above	0.311*	0.129, 0.751	0.0090	19.897**	3.175, 124.685	0.0010
Experience of driver						
0–5 years	1					
5–10 years	0.014**	0.007, 0.027	< 0.0001	0.0005	2.698E-17, 1.03E+10	0.6290
above 10	0.101**	0.057, 0.176	< 0.0001	0.0348	3.236E-11, 37426516	0.7520
Vehicle-driver ownership						
Employee	1					
Owner	0.822	0.289, 0.429	0.7130	0.096**	0.028, 0.336	0.0000
Others	1.168	0.607, 2.246	0.6420	54.833	0.007, 404707.6	0.3780
Education level of driver						
Primary/below	1					
High school	1.029	0.975, 1.087	0.2940	0.980	0.224, 4.287	0.9790
Above high school	1.003	0.573, 1.753	0.9920	0.096	3.8007E-07, 24074.54	0.7110
Type of vehicle						
Taxi-minibuses (up to 12 seats)	1					
Automobile	8.642**	2.764, 27.023	< 0.0001	131.837	1.763E-07, 9.85E+10	0.6400
pick up (up to 10 quintals)	0.379	0.085, 1.679	0.2010	747.915	1.606E-06, 3.48E+11	0.5160
Cargo (11–40 quintals)	0.453	0.094, 2.182	0.3240	1.28e^+7^	4.686E-15, 3.5E+28	0.5160
cargo (41–100 quintals)	1.059	0.197, 5.692	0.9460	2.29e^+11^	2.759E-08, 1.9E+30	0.2390
Cargo with trailer (up to 400 quintals)	3.372	0.506, 22.477	0.2090	5.20e^+16^	2.767E-06, 9.79E+38	0.1410
Buses (13–45 seat)	4.614	0.626, 33.989	0.1330	2.44e^+20^	1.656E-41, 3.6E+81	0.5140
Buses (above 46 seat)	6.499	0.863, 48.938	0.0690	2.65e^+20^	1.615E-41, 4.35E+81	0.5130
Others	4.328	0.584, 32.073	0.1520	5.37e^+19^	2.503E-42, 1.15E+81	0.5280
Vehicle length of service						
0–5 years	1					
5–10 years	2.484*	1.194, 5.169	0.0150	66.691	4.623E-36, 9.62E+38	0.9230
above 10 years	2.639*	1.268, 5.497	0.0090	0.978	0.241, 3.975	0.9750
Road condition						
Wet	1					
Dry	4.739**	2.400, 9.356	< 0.0001	91.464**	46.138, 181.317	0.0000
Muddy	0.937	0.829, 1.058	0.2930	0.158	0.015, 1.652	0.1230
Time of accident						
Afternoon	1					
Morning	0.709	0.179, 2.806	0.6240	0.736	0.020, 27.153	0.8680
Evening	0.472**	0.369, 0.603	< 0.0001	0.134*	0.020, 0.609	0.0090
Night	0.480**	0.370, 0.623	< 0.0001	0.009*	0.0002, 0.411	0.0160
Location of accident						
Central zones	1					
Western zones	0.275**	0.167, 0.455	<0.0001	0.013*	0.0007, 0.224	0.0030
South west zones	0.991	0.779, 1.259	0.9400	0.044**	0.010, 0.192	0.0000
Eastern zones	0.810	0.608, 1.081	0.1520	0.072**	0.021, 0.253	0.0000
South east zones	0.624*	0.410, 0.950	0.0280	0.074*	0.014, 0.400	0.0030
Environment of accident						
Factory place	1					
Residence place	108.506**	13.725, 857.798	< 0.0001	58.843	5.795E-22, 5.97E+24	0.8800
Commercial place	0.475	0.109, 2.061	0.3200	174.960**	25.519, 1199.53	0.0000
Religious place	0.378	0.116, 1.235	0.1070	23.079*	1.397, 381.369	0.0280
Entertainment place	0.439	0.067, 2.859	0.3890	63.152	5.150E-19, 7.74E+21	0.8610
School place	0.707	0.381, 1.313	0.2720	.0005	1.677E-17, 1.78E+10	0.6360
Hospital place	23.789*	3.038, 186.298	0.0030	48.039	3.59E-23, 6.43E+25	0.8910
Rural village area	0.152*	0.028, 0.810	0.0270	1.28e^-09^	1.55E-21, 1064.976	0.1440
Out of village in rural area	0.268	0.064, 1.128	0.0730	1.41e^-07^	1.6229E-19, 121649.6	0.2610
Government office area	129.606**	16.448, 1021.263	<0.0001	64.255	2.2128E-22, 1.87E+25	0.8800
Road Pavement						
Good asphalt	1					
Defected asphalt	0.947	0.441, 2.035	0.8900	0.00001*	3.098E-09, 0.039	0.0060
Gravel	0.497	0.220, 1.125	0.0930	3.15e-06	8.161E-19, 12174328	0.3920
Site clearing	0.567	0.253, 1.267	0.1670	6.12e-06	7.081E-19, 52909317	0.4300
Type of accident						
Collision between vehicles	1					
Collision between vehicle and pedestrian	1.408	0.861, 2.303	0.1730	6.872	1.9758E-08, 2.39E+09	0.8480
Collision between vehicle and animal	1.298	0.784, 2.147	0.3110	0.813	0.109, 6.095	0.8410
Vehicle upside down	5.560**	2.506,12.336	0.0000	14.758	2.929E-08, 7.44E+09	0.7920
Others	1.453*	1.009, 2.092	0.0440	0.485	0.105, 2.239	0.3530
Accident cause						
Not given priority to pedestrian	1					
Over load	0.749	0.359, 1.559	0.4390	7.76e-09	2.3157E-39, 2.6E+22	0.6030
Over speed	0.401	0.077, 2.096	0.2790	1.24e-13**	8.178E-17, 1.89E-10	0.0000
Turning illegal position	0.454*	0.226, 0.913	0.0270	7.24e-14*	4.574E-23, 0.000115	0.0050
Steering problem	0.693	0.249, 1.925	0.4820	1.87e-21	2.160E-84, 1.61E+42	0.5190
Brake problem	0.726	0.219, 2.411	0.6010	6.13e-18	1.428E-40, 262663.8	0.1360
Release of tire	0.708	0.256, 1.963	0.5070	6.56e-22	3.312E-85, 1.3E+42	0.5120
Following not keeping distance between	0.699	0.415, 1.179	0.1800	1.31e-07	5.887E-19, 29339.79	0.2350

** Significant p-value < 0.001

* significant p-value < 0.05.

The covariates age of drivers, driving experience, vehicle type, vehicle service years, road condition, time of the accident, location, environment of the accident, type of accident, and cause of the accident were statistically significant predictors of experiencing human deaths. The multivariable HP analysis presented in Table 3, illustrated that drivers aged 30–50 years old were associated with a 71% (adjusted odds ratio (AOR) = 0.289; 95% confidence interval (CI): 0.175, 0.479) decreased risk of experiencing human death per accident due to road traffic accident, whereas those drivers aged at least 50 years old was associated with a 69% decreased risk of experiencing human deaths (AOR = 0.311; 95%CI: 0.129, 0.751)as compared to drivers 18–30 years old holding all other variables in the model constant. The driving experience of drivers was found inversely statistically significant. The driving experience of 5–10 years associated with a98% (AOR = 0.014; 95%CI: 0.007, 0.027) lower risk experiencing human deaths per road traffic accident, likewise driving at least 10years of experience had an 89.9% (AOR = 0.101; 95%CI: 0.057, 0.176) lower risk of experiencing human deaths due to road traffic accidents compared to the driving experience of lower than 5 years.

As vehicle type was concerned, automobiles were 8.642(AOR = 8.642; 95%CI: 2.764, 27.023) times more likely to experience human deaths per road traffic accident compared to taxi minibus with up to 12 seats. Moreover, vehicles served 5–10 years (AOR = 2.484; 95%CI: 1.194, 5.169) and at more than 10 years (AOR = 2.639; 95%CI: 1.268, 5.497) was associated with a higher risk of experiencing human deaths per road traffic accident as compared to vehicles served not more than five years. Furthermore, the expected number of human deaths per road traffic accident was 4.739(AOR = 4.739; 95%CI: 2.400, 9.356) times higher for driving on wet roads as compared to dry roads. The average number of human deaths per road traffic accident occurred during the evening and night time was statistically significantly lower than the number of human deaths occurred in the afternoons (AOR = 0.472; 95%CI: 0.369, 0.603) and (AOR = 0.480; 95%CI: 0.370, 0.623), respectively.

The result in Table 3, also illustrated that as geographical regions were concerned, the western zone, which includes the four Wollega zones was associated with 72% (AOR = 0.275; 95%CI: 0.167, 0.455) decreased risk expected human deaths while the south Eastern zone, including Arsi and Guji, was associated with 36% (AOR = 1.363; 95%CI:1.084, 1.887) and 1.380 (AOR = 0.624; 95%CI: 0.410, 0.950) increases the risk of expected human deaths compared to the special zones surrounding Finfinne. Furthermore, the results showed that the odds of experiencing human deaths per road traffic accident was higher among the accidents happened near residential (AOR = 108.506; 95%CI: 13.725, 857.798), government office areas (AOR = 129.606; 95%CI: 16.448, 1021.263) and around hospitals (AOR = 23.789; 95%CI: 3.038, 186.298) compared to those accidents happened near factories. But it was lower in the rural village areas (AOR = 0.152; 95%CI: 0.028, 0.810).

The result also illustrated that the vehicle upside down accident type (AOR = 5.560; 95%CI: 2.506, 12.336) and vehicle crash to inert (AOR = 1.453; 95%CI: 1.009, 2.092)was associated with experiencing a higher expected human deaths per road traffic accident compared collision between vehicles. In contrast, the number of human deaths per road traffic accident due to illegal turning was 54.5% (AOR = 0.454; 95%CI: 0.226, 0.913) lower as compared to the number of human deaths by accidents due to denying priority to pedestrians.

The second part of Table 3 provides estimated odds for the factor change in the odds of being in the zero counts group (no human death per road traffic accident faced) compared to the non-zero count group (at least one human death per road traffic accident). The driver’s age, vehicle-driver relationship, road condition, time of the accident, location of the accident, the environment of the accident, road pavement, and cause of the accident had significantly associated with the probability of being in the zero counts group. The odds of being in the zero counts group was 19.89 (AOR = 19.897; 95%CI: 3.175, 124.685) times more likely among drivers aged at least 50 years as compared to those aged18-30 years controlling other variables in the model. About the vehicle-driver relationship, the odds of being in the zero counts group was 0.096 (AOR = 0.096; 95%CI: 0.028, 0.336) times less likely among owner drivers as compared to employee drivers. In contrast, the odds of being in the zero count group was increased by a factor of 91.464(AOR = 91.464; 95%CI: 46.138, 181.317) for road traffic accidents on wet roads as compared to accidents on dry roads.

The time of the accident had a significant association with the odds of being in the zero counts group. For instance, as compared to accidents occurred in the afternoon, the odds of being in the zero counts group reduced by 86.6% (AOR = 0.134; 95%CI: 0.020, 0.609), and 99% (AOR = 0.009; 95%CI: 0.0002, 0.411) for accidents happening in the evening and night time, respectively. Similarly, as the location of accidents was concerned the odds of being in the zero counts, was reduced by 98.7% (AOR = 0.013; 95%CI: 0.0007, 0.224), 95.6% (AOR = 0.044; 95%CI: 0.010, 0.192),92.8% (AOR = 0.072; 95%CI: 0.021, 0.253), and 92.6% (AOR = 0.074; 95%CI: 0.014, 0.400), for western zones, southwestern zones, eastern zones and southeastern zones respectively so as compared to the central zones. On the other hand, the odds of being in the zero counts group was 174.96 (AOR = 174.96; 95%CI: 25.519, 1199.53) and 23.079(AOR = 23.079; 95%CI: 1.397, 381.369), times more likely for road traffic accidents happened at commercial areas and worship areas respectively, as compared to accidents happened around factory areas. The odds of being in the zero counts group were reduced by 99% of road traffic accidents at road pavements on defected asphalt roads as compared to those on road pavements on good asphalt roads holding all other variables in the model constant. Whereas, the odds of being in the zero counts group was reduced by 99.9% of road traffic accidents due to over-speeding as compared to those due to denying priority to the pedestrian.

## Discussion

This study was carried out to identify the major factors associated with the number of human deaths per road traffic accident based on Oromia region road traffic accident data. The total number of accidents from July 2016 to July 2017 was included in the study. The hurdle Poisson regression model was found as the most appropriate model from other possible count models.

Driversaged18-30 had a higher attribute to the number of human deaths per road traffic accident as compared to those driversaged30 years or higher. This was congruent to previous investigations in Addis Ababa [13,19] and Bahir Dar city [12]. The driving experience of lower than 5 years was related to a higher human death per road traffic accidents when contrasted with the individuals who have over 5 years of driving experience. Similarly, prior studies reported that the smaller driving experience a higher probability of experiencing human deaths per accident [16–18]. Among vehicle types, the numbers of deaths due to road traffic accidents happened by automobiles were significantly greater than other vehicle types. This finding is consistent with the results of Towelde [13] and Ahmed [34]. Consistent with the findings by Fikadu [20]; older vehicles were associated with accidents resulting in a higher number of human deaths per road traffic accidents as compared to accidents due to vehicles served for less than 5 years. The possible reason for this might be attributed to the poor maintenance of vehicles.

Accident time was another factor significantly associated with the number of human deaths per road traffic accident. The lower number of human deaths per road traffic accident took place in the evenings and night times as compared to afternoons. This finding is similar to the findings of previous studies [20,21,43], and this variation in road traffic accidents by times of the day reflects variations in traffic volumes. Furthermore, the higher number of human deaths per accident happened in the residential areas, hospital areas, and government office areas, whereas, the fewer number of human deaths per accident happened in rural villages than near factory areas. This finding is congruent with the results of previous studies [21,44]. Consistent with the findings of Getahun [19], vehicle upside down and other accident types such as vehicle down, vehicle crash to inert were associated with the increased number of human deaths per road traffic accident as compared to the number of human deaths due to collision between vehicles.

The number of human deaths per road traffic accident that happened due to turning in an illegal direction was lower as compared to the number of deaths that happened due to drivers denying priority to pedestrians. It was observed that the number of human deaths per road traffic accident due to denying priority to pedestrians was more than the number of deaths by accidents due to other causes. This was consistent with the results of studies done in central Ethiopia [20] and Bahir Dar [12]. Similar to a study in Dire Dawa [22], the cause of accidents about 80% of car accidents were attributed to driver faults among which denying priority for a pedestrian was the leading.

## Limitation of the study

This study depends on secondary information. However, the reliability of the data related to the driver’s age, vehicle service years, and driving experience was questionable as these were collected by interviewing the drivers themselves. Moreover, some very useful data for variables such as the utilization of seat belts and helmets, the use of alcohol, and talking on a mobile phone while driving were not available.

## Conclusion

Fatal RTA was attributed to the younger age of the driver, drivers lack of sufficient experience, type of vehicle (automobile), vehicle serviced for long years, driving on a wet road, driving on the afternoon, driving near/around residential places, vehicle to driver’s relation, type of accident (vehicle upside down) and turning to illegal positions. Special attention should be needed for younger drivers, less experienced drivers, old vehicles, driving near residential areas, driving automobiles, and driving in the afternoon. Thus, the regional traffic police should give special attention to control used vehicles and old vehicles on the road, and controlling traffic systems near residences and workplaces to reduce the number of human deaths per road traffic accident. Finally, further study should be conducted to identify mechanisms for addressing the causes of road traffic accidents and consequences in the study area that will show the seasonal variation.

## Supporting information

S1 Data (XLSX)

## Abbreviations

AIC: Akaike Information Correction
AOR: Adjusted odds ratio
BIC: Bayesian Information Correction
CI: Confidence Interval
GDP: Gross Domestic Product
GLMs: Generalized Linear Models
HNB: Hurdle Negative Binomial
HP: Hurdle Poisson
NB: Negative Binomial
RTA: Road Traffic Accidents
SD: Standard Deviation
ZIP: Zero-Inflated Poisson
ZINB: Zero-Inflated Negative Binomial
